# Efficacy and safety of Zaoren Anshen capsules in the treatment of insomnia: A meta-analysis of randomized controlled trials

**DOI:** 10.1097/MD.0000000000019033

**Published:** 2020-02-07

**Authors:** Bo-Wei Chen, Jian Yi, Bei Sun, Ping Jia, Wen-Jiang Zhang, Bai-Yan Liu

**Affiliations:** a The First Affiliated Hospital, Hunan University of Chinese Medicine, Changsha, Hunan, P.R. China; b Yiyang Medicine College, Yiyang, Hunan, P.R. China.

**Keywords:** Chinese patent medicine, efficacy and safety, insomnia, meta-analysis

## Abstract

**Background:**

Zaoren Anshen capsules (ZRAS) have been widely used to treat patients with insomnia. However, the efficacy and safety of ZRAS for insomnia treatment is not entirely clear. Therefore, it is necessary to clarify the effect of ZRAS for the treatment of insomnia by a systematic meta-analysis.

**Methods:**

We searched PubMed, EMBASE, Web of Science, Cochrane Library, Chinese National Knowledge Infrastructure (CNKI), and WanFang databases and performed a manual search to retrieve relevant articles (available through January 2019) describing randomized controlled trials (RCTs) of ZRAS for the treatment of insomnia. The quality of the selected articles was assessed with the Cochrane risk-of-bias tool. A meta-analysis of the selected articles was performed with RevMan 5.3 software.

**Results:**

A total of 13 articles including 1175 patients were included in the study. Overall, our results showed that ZRAS was slightly higher than that of the conventional Western medicine for insomnia in terms of clinical efficacy rate; but there was no statistical difference between the 2 groups (relative risk [RR] = 1.03, 95% confidence interval [CI] = [0.97, 1.09], *P* = .34). However, it should be noted that ZRAS treatment causes far fewer adverse reaction than treatment with conventional Western medicine (RR = 0.20, 95% CI = [0.14, 0.28], *P* < .00001).

**Conclusion:**

Our results suggested that ZRAS is an effective and safe treatment for insomnia, especially in adverse reaction. However, multi-regional and well-designed RCTs studies are needed in the future to validate the results.

## 1 Introduction

Insomnia is a chronic and recurrent or persistent sleep disorder. Typical symptoms include trouble sleeping, daytime cognitive dysfunction, and autonomic dysfunction.^[1]^ Epidemiological studies show that the prevalence rate of insomnia is 10% to 20%, and this rate increases significantly with age and has severe physical and mental consequences. Therefore, insomnia must be actively treated once diagnosed.^[2]^

Currently, the main Western treatments for insomnia include non-drug therapy and drug therapy.^[3]^ Non-drug therapy is recommended as first-line therapy and includes behavioral intervention therapy, music therapy, Chinese massage, and Swedish massage. However, in clinical practice, it may be difficult to implement non-drug therapy due to several factors. As a result, it is generally recommended to combine non-drug therapy and drug therapy.^[4]^ Furthermore, the use of conventional Western medicine for insomnia treatment has been gradually shown to have some shortcomings in clinical practice, such as residual sedative effects, dizziness, and fatigue on the next day following medication.^[5]^ Moreover, long-term use of Western medicine may lead to drug-dependent sleep-related behavioral disorders and rebound insomnia after drug discontinuation, causing certain concerns among patients. Therefore, it is necessary to explore new treatment options.

Studies have demonstrated the efficacy of traditional Chinese medicine (TCM).^[6]^ Since ancient times, TCM physicians have gained significant clinical experience in treating insomnia. Syndrome differentiation is the essence of TCM treatment. TCM treatments such as herbs and acupuncture may cause fewer side effects than but are as effective as conventional Western medicine.^[7]^ Based on syndrome differentiation, TCM physicians often prescribe formulations including Semen Ziziphi Spinosae, *Schisandra chinensis*, *Salvia miltiorrhiza*, *Rhizoma anemarrhenae*, and *Polygala tenuifolia* to treat insomnia,^[8]^ which are usually immediately effective when used properly.

Due to the constant development of TCM research and continuous improvement in pharmaceutical processes, many TCM preparations, including Zaoren Anshen capsules (ZRAS), manufactured to Western medicine standards have been developed for the treatment of insomnia.^[9]^ ZRAS includes 3 TCM ingredients, Semen Ziziphi Spinosae, *S chinensis*, and *S miltiorrhiza*. It is widely used in clinical practice. Currently, most studies on the efficacy of ZRAS are reported in the Chinese language, and no comprehensive or systematic evidence is available to validate its clinical efficacy for insomnia treatment. In this study, we retrieved eligible randomized controlled trials (RCTs) to perform a meta-analysis of the treatment outcomes of the use of ZRAS for insomnia.

## 2 Methods

This study complied with Preferred Reporting Items for Systematic Reviews and Meta-analyses (PRISMA) Statement.^[10]^ All analyses were based on published studies, therefore no ethical approval and patient consent are required.

### 2.1 Search strategy

We searched databases including WanFang, China National Knowledge Infrastructure (CNKI), Web of Science, PubMed, EMBASE, and the Cochrane Library for relevant articles available through January 2019. We used keywords consisting of: (“Zaoren Anshen capsules” OR “ZRAS”) AND (“insomnia” OR “sleep disorder”) AND (“randomized controlled trial” OR “randomized”). We also manually searched conference proceedings.

### 2.2 Inclusion criteria

Included studies must met the following criteria: types of articles: double-blind or single-blind RCTs and semi-RCTs using an allocation concealment method; patients enrolled: patients were diagnosed according to the Chinese Classification of Mental Disorders (CCMD-3)^[1]^ or the Guidelines for Clinical Research of New Traditional Chinese Medicine^[11]^; intervention: ZRAS; control measure: conventional Western medicine consisting of benzodiazepines (BZDs) or non-benzodiazepines (NBZDs); outcome measures: efficacy, Pittsburgh Sleep Quality Index (PSQI)^[12]^ (to assess sleep quality), and adverse reactions; and efficacy criteria: highly effective: time to fall asleep <30 minutes, sleep time extended by ≥2 hours, patients reported feeling good, PSQI ≤7; effective: time to fall asleep 30 to 45 minutes, sleep time extended by ≥1 hour, patients felt significantly better, PSQI reduced by >30%; no response: time to fall asleep >45 minutes, sleep time extended by <1 hour, patients did not feel better, PSQI reduced by <25%.

### 2.3 Exclusion criteria

Exclusion criteria as follows: reviews, case reports, duplicate publications; non-human studies, such as animal or laboratory studies; non-RCTs study; incomplete raw data or measures.

### 2.4 Data extraction

Two evaluators independently searched, selected, and organized the articles. Any disagreement was resolved through discussion with a third evaluator. Extracted data as follows: publication time, author, sample size, age group, sex, intervention method, treatment duration, outcome index, and adverse reaction. The selected articles were evaluated according to the Cochrane System Evaluator's Manual.

### 2.5 Statistical analysis

RevMan 5.3 software (Copenhagen: The Nordic Cochrane Centre, The Cochrane Collaboration, 2014.) was used for this meta-analysis, and a heterogeneity test was performed. A chi-square test was performed to analyze any heterogeneity between studies. A random effects model was used in the case of significant heterogeneity (*P* < .10, *I*^2^ > 50%); otherwise a fixed effects model was used (*P* > .10, *I*^2^ < 50%). The subgroup analysis was used based on drug type in controls. For binary categorical variables, the relative risk (RR) and 95% confidence interval (CI) are given; for continuous variables, the mean difference (MD) and 95% CI are given. A funnel plot was used to analyze the potential publication bias. *P*-value <.05 was considered statistically significant.

## 3 Results

### 3.1 Search results

**Figure 1:**
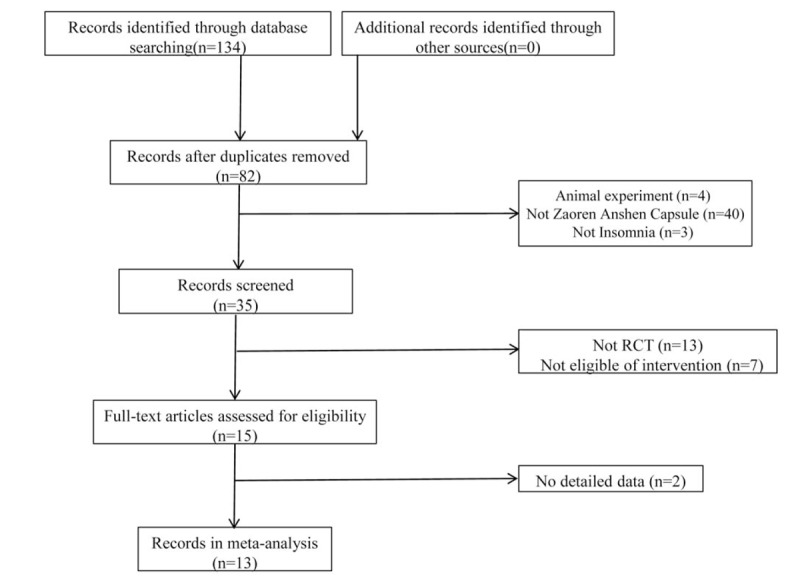
Study selection process for the meta-analysis.

A total of 134 articles were initially retrieved. Three independent evaluators screened the articles according to rigorous criteria and finally selected 13 articles that included 1175 patients.^[13–25]^Figure 1 shows the screening process, and Table 1 listed the basic characteristics of included studies.

**Table 1:**
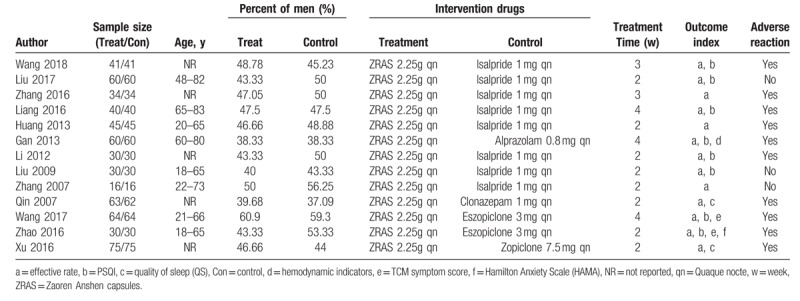
Characteristics of the studies included in the meta-analysis.

### 3.2 Quality evaluation

**Figure 2:**
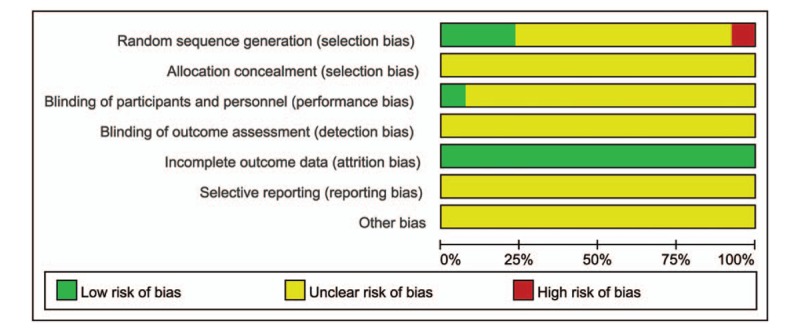
Risk of bias graph.

**Figure 3:**
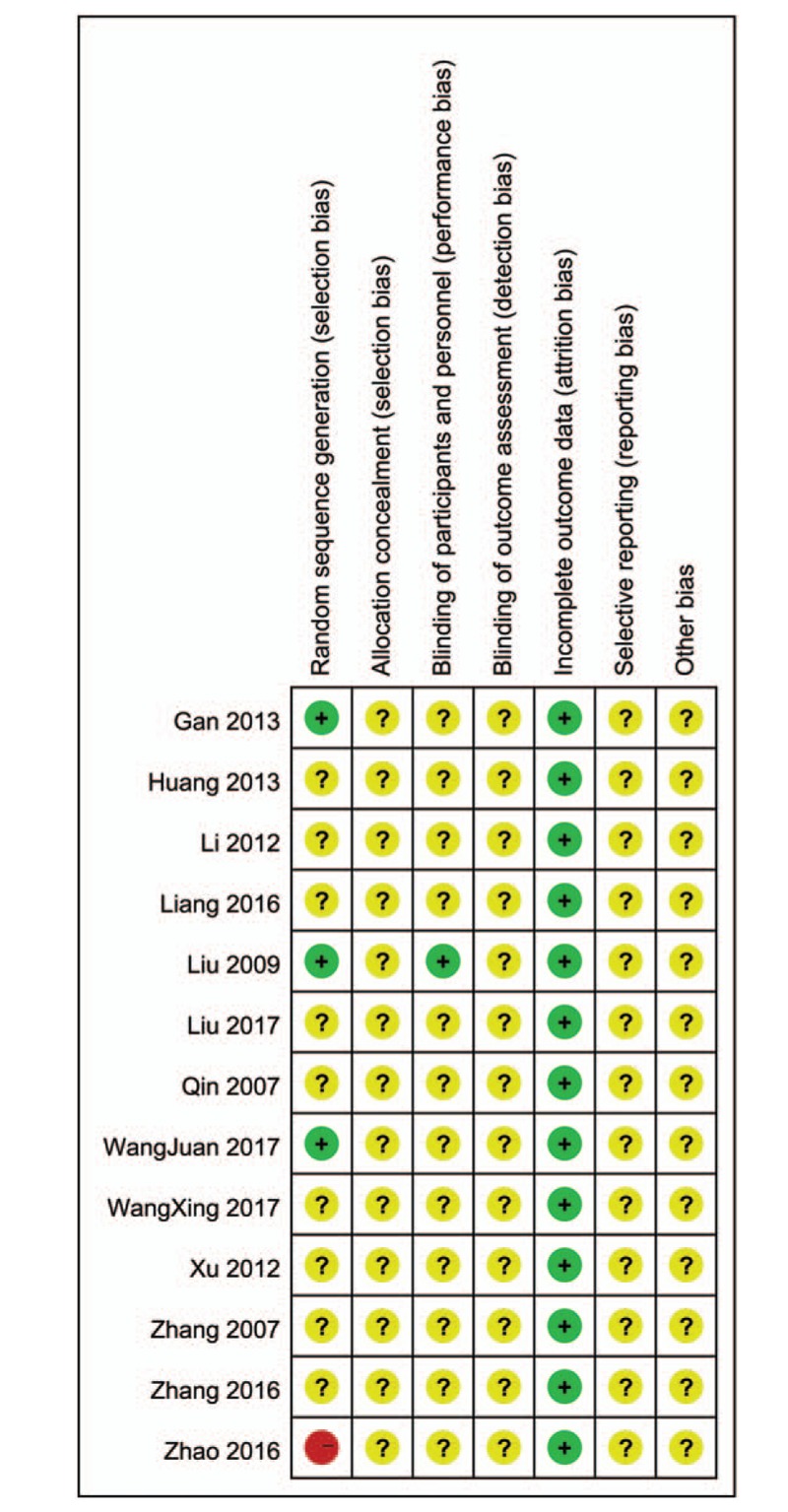
Risk of bias summary.

All 13 studies were randomized. Among them, a random number sheet was used in 3 studies,^[13,18,23]^ an incorrect allocation method was used in 1 study,^[25]^ and “randomization” was mentioned but details were not provided in the remaining studies. One study included a placebo group and was conducted in a double-blind manner.^[18]^ None of the studies discussed allocation concealment. All studies had complete data; none of the studies were evaluable for selective reporting or other bias. According to Cochrane risk-of-bias tool, the quality of the selected articles was moderate, and a risk of bias was present in Figs. 2 and 3.

## 4 Meta-analysis results

### 4.1 Clinical efficacy

**Figure 4:**
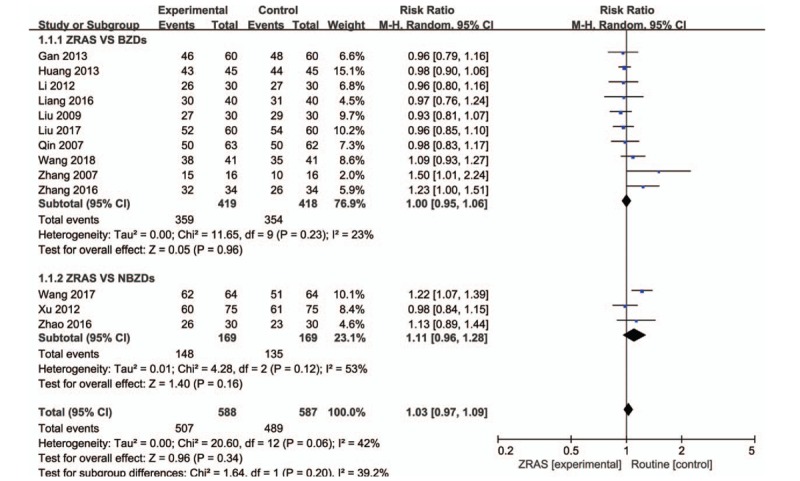
Comparison of the clinical efficiency between ZRAS and conventional Western medicine in insomnia patients using a random effect model and subgroup analysis of insomnia intervention conditions. ZRAS = Zaoren Anshen capsules.

The efficacy was evaluated according to standardized criteria and rated as highly effective, effective, and no response. The first 2 ratings were considered clinically effective, and the third was considered ineffective. A heterogeneity test of the 13 studies (1175 patients) was conducted (*P* = .06, *I*^2^ = 42%), so the fixed effects model was applied. The results showed that the clinical efficacy rate of the ZRAS group (86.2%) was slightly higher than that of the conventional Western medicine (83.3%) for the treatment of insomnia; but there was no statistical difference between the 2 groups (RR = 1.03, 95% CI = [0.97, 1.09], *P* = .34) (Fig. 4).

We further performed a subgroup analysis per drug type in the control group. Ten studies^[13–19,21,23,24]^ used BZDs in the control group, with a pooled RR = 1.00, 95% CI = [0.95, 1.06], and *P* = .96, indicating no significant difference. Three studies used NBZDs in the control group, with a pooled RR = 1.11, 95% CI = [0.96, 1.28], and *P* = .16, indicating no significant difference.

### 4.2 PSQI score

**Figure 5:**
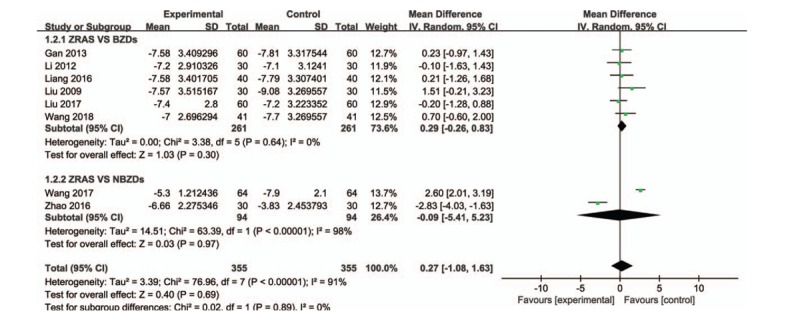
Comparison of the PSQI between the ZRAS-treated and control insomnia patients using a random effect model. PSQI = Pittsburgh Sleep Quality Index, ZRAS = Zaoren Anshen capsules.

The PSQI was used to evaluate the sleep quality of 710 patients in 8 studies.^[13,15–18,20,21,25]^ A heterogeneity test was conducted (*P* < .00001, *I*^2^ = 91%), and so random effects model was used to pooled analysis. The results indicated that there was no statistically significant association between ZRAS group and Western medicine group in the PSQI score of insomnia (MD = 0.27, 95% CI = [−1.08, −1.63], *P* = .69) (Fig. 5).

### 4.3 Adverse reactions

**Figure 6:**
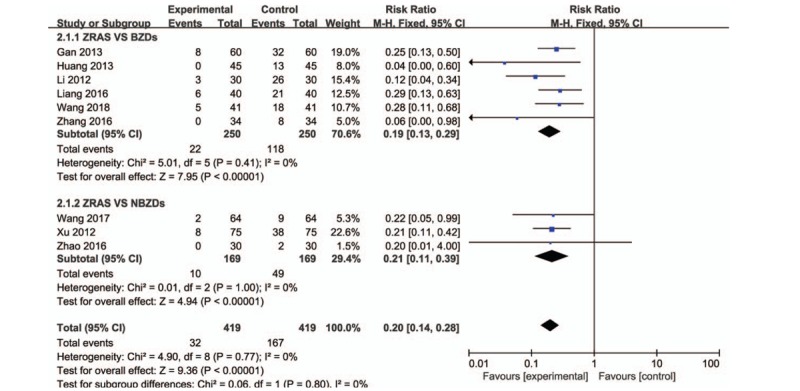
Comparison of adverse reactions between ZRAS and conventional Western medicine for the treatment of insomnia. ZRAS = Zaoren Anshen capsules.

Nine studies (838 patients)^[13–16,20–23,25]^ described adverse reactions, including dizziness, drowsiness, and fatigue (Fig. 6). A heterogeneity test was conducted (*P* = .77, *I*^2^ = 0%), and so the fixed effects model was conducted to pooled analysis. The results demonstrated that the adverse reactions rate of the ZRAS group (7.64%) was significantly lower than that of the conventional Western medicine (39.86%) for the treatment of insomnia (RR = 0.20, 95% CI = [0.14, 0.28], *P* < .00001) (Fig. 4).

### 4.4 Publication bias

**Figure 7:**
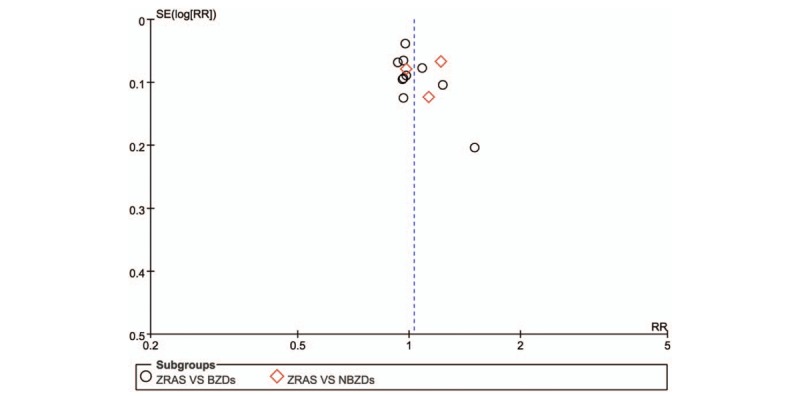
Funnel plot of the comparison of publication bias between ZRAS and routine Western medicine treatment. ZRAS = Zaoren Anshen capsules.

Figure 7 shows the funnel plot for publication bias. An inverted funnel indicates no publication bias, while an incomplete or asymmetrical funnel indicates certain publication bias. Figure 7 shows an inverted but asymmetrical funnel, indicating the presence of publication bias, which may be related to low quality, small sample size, and selective reporting of the included articles.

The funnel plot was used for potential publication bias evaluation, and the results are shown in Fig. 7. The shape of the funnel plots no apparent asymmetry, indicating that there was no significant publication bias in our study.

## 5 Discussion

This study represents the first systematic meta-analysis of ZRAS for the treatment of insomnia. This study included 1175 patients from 13 studies and analyzed the clinical efficacy, PSQI, and adverse reactions. This meta-analysis showed that ZRAS was slightly higher than that of the conventional Western medicine for insomnia in terms of clinical efficacy rate, both in the overall analysis and subgroup analysis; but there was no statistical difference between the 2 groups. This means ZRAS did not significantly improved the clinical efficacy as compared with conventional Western medicine. However, it should be noted that ZRAS treatment causes far fewer adverse reactions than treatment with conventional Western medicine (*P* < .00001). In summary, ZRAS is safe and effective for insomnia treatment and should be more widely used in clinical practice.

TCM considers insomnia a syndrome of “difficulty in sleeping,” which is often caused by emotional disorders, improper diet, and imbalance between labor and rest, resulting in malaise and thus an inability to properly sleep. The 3 main TCM ingredients of ZRAS are Semen Ziziphi Spinosae, *S chinensis*, and S miltiorrhiza, which have proven effects of nourishing blood and soothing the nerves and are mainly clinically used to treat symptoms such as insomnia, amnesia, and dysphoria.

Sleep disorders are associated with many factors, including physical factors, mental factors, environmental factors, physical illnesses, and drugs used to treat other illnesses. Studies show that neurotransmitters such as 5-hydroxytryptamine (5-HT), γ-aminobutyric acid (GABA), acetylcholine (Ach), and dopamine (DA) are closely related to sleep, and any disturbance of these neurotransmitters may cause insomnia.^[26]^ Modern pharmacological studies show that Semen Ziziphi Spinosae, one of the ingredients of ZRAS, contains active substances such as jujuboside, total flavonoids, and total alkaloids that have sedative and hypnotic effects and can extend sleep time.^[27]^ These effects may be related to the regulation of 5-HT, GABA, and glutamic acid (Glu).^[28–30]^*S chinensis* has sedative, hypnotic, and anti-anxiety effects on the central nervous system, and such effects may be related to the Glu level.^[31]^*S chinensis* also improves cognitive function and has anti-oxidative effects.^[32,33]^*S miltiorrhiza* dilates blood vessels and improves capillary permeability, thereby improving the microcirculation and resisting hypoxia in the brain.^[34]^*S miltiorrhiza* also has certain antidepressant effects.^[35]^ Thus, the ingredients of ZRAS have a wide range of pharmacological effects, demonstrating the characteristics of TCM compounds, such as multiple components, multiple targets, and synergistic roles in achieving therapeutic effects.

This meta-analysis has the following limitations. The PSQI analysis showed significant heterogeneity, which may be related to the insufficient number of studies, small sample size, and different intervention conditions of the included studies. The quality of most studies was moderate, none of the studies discussed allocation concealment, and only a few studies clearly described double-blind measures. Moreover, none of the studies mentioned follow-up. As a result, the long-term outcome was unknown, and thus it is impossible to evaluate the medium-to-long-term efficacy of ZRAS for insomnia treatment. Finally, all included articles are written in Chinese, and all studies were conducted and published in China, indicating certain publication bias. Therefore, multi-regional, large-scale, high-quality RCTs are needed in the future to validate the results.

## 6 Conclusion

To sum, our results suggested that ZRAS is an effective and safe treatment for insomnia, especially in adverse reaction. However, high-quality, well-designed, multi-center RCTs are needed in the future to provide more reliable evidence.

## Author contributions

**Conceptualization:** Bai-Yan Liu.

**Data curation:** Bo-Wei Chen.

**Formal analysis:** Bo-Wei Chen, Bai-Yan Liu.

**Funding acquisition:** Jian Yi, Wen-Jiang Zhang, Bai-Yan Liu.

**Investigation:** Jian Yi, Wen-Jiang Zhang, Bai-Yan Liu.

**Methodology:** Bo-Wei Chen, Jian Yi, Ping Jia.

**Project administration:** Jian Yi, Ping Jia, Wen-Jiang Zhang.

**Resources:** Wen-Jiang Zhang.

**Software:** Jian Yi, Bei Sun, Ping Jia.

**Supervision:** Bo-Wei Chen, Bei Sun, Ping Jia.

**Writing – original draft:** Bei Sun, Bo-wei Chen.

**Writing – review & editing:** Bo-Wei Chen.
